# 

**Published:** 1875-10

**Authors:** 


### Editorial and Miscellaneous.

*To our Delinquent Subscribers.*

Simultaneously with the mailing of the August number of this journal, we furnished each delinquent subscriber a statement of his account up to the first of January, 1876, requesting his early attention to the matter. Some of these have responded, while many have, up to the present, failed to notice our request. It is certainly absurd to presume that any physician in the regular practice is unable to raise the small sum of three dollars in thirty days, and we will not indulge the suspicion that any of the gentlemen now in arrears for subscription are so destitute of honor as to intentionally avoid the payment due. There is, therefore, but one key with which to solve the problem of their failure to respond, and that is the fact of negligence which so cruelly afflicts many good men, or a forgetfulness that while a little more excusable is none the less an evil to us.

The preparation of the Journal for the profession, if saved from these delays and losses, would indeed be a pleasure, but we cannot afford the time required to manage its business or financial details, unless promptness should characterize our subscribers. We, therefore, make a personal appeal to each of these, and request him no longer to neglect or forget the obligation which he has assumed, but to wake up and act at once in remitting his dues. If any should treat this appeal with indifference, we shall be compelled, and pledge our honor, to send his account for collection through such a channel as will incur additional expense.

### LIST OF PAYMENTS.

| | |
|---|---|
| Dr. J. Hatch, Va | $3 00 |
| Dr. T. L. Quillian, Ala. | 3 00 |
| Dr. Robert W. Lovett, Ga | 3 00 |
| Dr. John H. Howard, Tenn | 2 00 |
| Dr. W. H. Fitch, S. C | 3 00 |
| Dr. H. Alison, La | 3 00 |
| Dr. J. B. Wilson, N. C | 3 00 |
| Dr. J. N. Taylor, La | 3 20 |
| Dr. J. J. Gage, Miss | 1 00 |
| Dr. Virgil Snody, Ala | 2 50 |
| Dr. M. P. Deadwyler, Ga | 3 00 |
| Dr. L. E. Kirkman, N. C | 3 00 |
| Dr. J. D. Moor, Ga | 3 00 |
| Dr. N. B. Warren, Miss | 3 00 |
| Dr. W. T. Mathis, Tenn | 3 00 |
| Dr. A. Dyer, Ind | 3 00 |
| Dr. E. P. Booth, Texas | 3 00 |
| Dr. J. H. Shelton, N. C | $3 00 |
| Dr. John H Henry, Ala | 3 00 |
| Dr. J. R. Johnson, Ala | 1 50 |
| Dr. S. E. Winnemore, Ala | 3 00 |
| Dr. James W. Howard, Oregon | 3 00 |
| Dr. F. M. Bledsoe, Ga | 2 50 |
| Dr. J. H. Cook, N. C | 3 00 |
| Dr. Wm. Gould, N. Y | 3 00 |
| Dr. E. W. Covington, Ga | 1 25 |
| Dr. John W. Starr, Ga | 2 00 |
| Dr. J. B. Baily, Miss | 3 00 |
| Dr. Thos. A. Cook, La | 3 00 |
| Dr. G. A. Whitney, Oregon | 3 00 |
| Dr. D. R. Fox, La | 3 00 |
| Dr. Alex. J. Kalb, La | 2 50 |
| Dr. A. B. Calhoun, Ga | 3 00 |

### COLLABORATORS AND CONTRIBUTORS.

GEORGIA.

James A. Long, M.D.
W. L. M. Harris, M.D.
H. L. Battle, M.D.
W. C. Musgrove, M.D.
L. B. Bouchelle, M.D.
Thomas Burdell, M.D.
E. H. W. Hunter, M.D.
V. S. Hitt, M.D.
J. E. Pope, M.D.
J. C. Wisdom, M.D.
W. W. Leake, M.D.
S. G. White, M.D.
W. H. Hall, M.D.
G. D. Case, M.D.
W. T. Grant, M.D.
J. M. Tules, M.D.
J. N. Cheney, M.D.
T. M. Shaw, M.D.
T. S. Mitchell, M.D.
H. B. Lee, M.D.
C. B. Nottingham, M.D.
W. L. Kirkpatrick, M.D.

KENTUCKY.

W. S. Taylor, M.D.
B. W. Stone, M.D.
Charles Kearns, M.D.

TEXAS.

S. M. Welch, M.D.
T. J. Heard, M.D.
L. R. Lincecum, M.D.

TENNESSEE.

J. D. Tucker, M.D.
F. A. Ramsey, M.D.
J. W. Hayne, M.D.
F. K. Bailey, M.D.

WEST VIRGINIA.

N. D. Tobey, M.D.

ALABAMA.

J. C. Knox, M.D.
Geo. F. Taylor, M.D.
J. B. Barnett, M.D.
S. M. Hogan, M.D.
J. T. Searcy, M.D.
A. C. Edwards, M.D.
M. W. Morton, M.D.
J. D. Rush, M.D.
J. J. Grace, M.D.

NORTH CAROLINA.

J. B. Hughes, M.D.
S. S. Satchwell, M.D.
A. B. Pierce, M.D.
H. Kelly, M.D.
Geo. A. Foote, M.D.
E. A. Anderson, M.D.
H. T. Bahnson, M.D.
John W. Booth, M.D.
R. S. Hallsey, M.D.

SOUTH CAROLINA.

E. T. McSwain, M.D.
G. M. Rivers, M.D.

OHIO.

J. Q. A. Hudson, M.D.

C. H. Tidd, M.D.

J. C. Bishop, M.D.

W. P. Wells, M.D.

NEW YORK.

Ely Van DeWarker, M. D.
C. C. F. Gay, M.D.
J. S. Bailey, M.D.

COLORADO.

Wm. R. Whitehead, M.D.

VIRGINIA.

F. Horner, Jr., M.D.
R. J. Preston, M.D.
J. S. Wharton, M.D.
Sam'l P. Ripley, M.D.
E. A. Drewry, M.D.
Rob. B. Tunstall, M.D.
W. F. Barr, M.D.
L. B. Anderson, M.D.
J. N. Upshur, M.D.
B. P. Reese, M.D.

MISSISSIPPI.

C. B. Gallaway, M.D.
Edward Lea, M.D.
S. V. D. Hill, M.D.
E. T. Henry, M.D.
T. P. Lockwood, M.D.
Robert Kells, M.D.
W. L. Lipscomb, M.D.
W. T. Beall, M,D.
A. G. Smythe, M.D.
J. M. Lewis, M.D.
J. H. Payne, M.D.
T. H. Mayo, M.D.
E. Cutter, M.D.

ARKANSAS.

A. D. Hodge, M.D.
J. K. Hodge, M.D.
A. W. Hobson, M.D.
C. D. Hodge, M.D.

INDIANA.

G. A. Dyer, M.D.
A. Patton, M.D.
Robert Robson, M.D.

ILLINOIS.

John H. Weir, M.D.

OREGON,

W. M. Cusick, M.D.

